# Economic impact of self-administered subcutaneous versus clinic-administered intravenous immunoglobulin G therapy in Alberta, Canada: a population-based cohort study

**DOI:** 10.1186/s13223-022-00735-6

**Published:** 2022-11-24

**Authors:** Bruce Ritchie, Karen J. B. Martins, Dat T. Tran, Heather Blain, Lawrence Richer, Scott W. Klarenbach

**Affiliations:** 1 Department of Medicine, University of Alberta, Edmonton, Canada; 2 Real World Evidence Unit, University of Alberta, Edmonton, AB Canada; 3 Institute of Health Economics, Edmonton, AB Canada; 4 School of Public Health, University of Alberta, Edmonton, AB Canada; 5 Alberta Precision Laboratories, Edmonton, AB Canada; 6 Department of Pediatrics, University of Alberta, Edmonton, AB Canada

**Keywords:** Immunoglobulin G, Subcutaneous, Intravenous, Home-based administration, Clinic-based administration, Administrative data, Real world evidence

## Abstract

**Background:**

Self-administered subcutaneous immunoglobulin G (SCIg) reduces nursing time and eliminates the need for treatment at ambulatory care clinics, as compared with clinic-based intravenously administered IgG (IVIg), and are therapeutically equivalent. Estimating the economic impact of self-administered SCIg versus clinic-administered IVIg therapy may guide treatment recommendations.

**Methods:**

A retrospective population-based cohort study using administrative data from Alberta was performed; those treated with IgG between April 1, 2012 and March 31, 2019 were included. Costs for medical laboratory staff and nursing time, as well as ambulatory care visits were considered. Univariate generalized linear model regression with gamma distribution and log link was used to compare cost ($CDN 2020) between SCIg and IVIg administration. Stratified analysis by age (≥ 18-years; < 18-years) was performed.

**Results:**

Among 7,890 (6,148 adults; 1,742 children) individuals who received IgG, the average administration cost per patient-year of self-administered SCIg was $5,386 (95% confidence interval [CI] $5,039, $5,734) lower than clinic-administered IVIg; per patient-year cost of self-administered SCIg was $817 (95% CI $723, $912) versus $6,204 (95% CI $6,100, $6,308) for clinic-administered IVIg. The per patient-year cost of self-administered SCIg was $5,931 (95% CI $5,543, $6,319) lower among adults and $3,177 (95% CI $2,473, $3,882) lower among children compared with clinic-administered IVIg. An estimated $31.0 million (95% CI $29.0, $33.0) in cost savings to the health system would be realised if 80% of individuals switched from clinic-administered IVIg to self-administered SCIg.

**Conclusions:**

Self-administered SCIg is substantially less costly from a health care payer perspective in Canada. Within this type of health system, switching to self-administered SCIg has the potential to reduce overall health care costs, lessen nursing burden, and may increase clinic-based capacity for others.

**Supplementary Information:**

The online version contains supplementary material available at 10.1186/s13223-022-00735-6.

## Background

Immunoglobulin G (IgG) administration was initially used to treat immunodeficiency diseases and over time the listed and off-label indications for IgG continue to increase [1–3]. IgG treatment can be administered by intravenous (IVIg) or subcutaneous (SCIg) infusion, with comparable effectiveness and a potentially more favourable safety profile for SCIg (less adverse events such as fever and severe headaches) compared with IVIg [4, 5]. The conventional method of IVIg infusion is typically performed on a monthly basis in an outpatient setting, whereas a newer alternative method of SCIg infusion can be self-administered by the patient at home. IVIg and SCIg may not be interchangeable in all cases, and thus it is not expected that home-based self-administered SCIg would completely replace clinic-based nurse-administered IgG (IVIg or SCIg); further there may be circumstance where patients are unable or unwilling to self-administer at home. However, while a considerable proportion of patients may be excellent candidates for self-administered SCIg, clinic-administered IVIg is currently used in the vast majority of patients requiring this therapy in Canada [6].

Some clinicians, hospitals, and provinces are embracing a self-administered SCIg therapy method, which may help reduce the burden on patients and the health care system. Also, a high willingness among patients to switch from clinic-administered to self-administered IgG therapy has been reported [7]. However, relatively few studies have compared the cost of SCIg with IVIg in the Canadian population; most have used modelling approaches [5, 8–11], and a few have conducted small studies using retrospective or prospective approaches [12, 13]. Although the findings of these studies showed self-administered SCIg was less costly compared with clinic-administered IVIg, there remains a need for real world population-based economic evaluation of these treatments. In this study, population-based data from Alberta, Canada was used to compare the health care cost of self-administered SCIg versus clinic-administered IVIg administration, and estimate the potential health care cost difference if various proportions of patients were transitioned from clinic-administered IVIg to self-administered SCIg therapy.

## Materials and methods

### Data source

Data was collected from the following databases from April 1, 2010 to March 31, 2019 unless otherwise stated. Over 99% of blood clinic facilities in Alberta electronically track incoming and outgoing IgG products; this data is collected and retained within the Alberta Health Services (AHS) provincial Lab Information System and contains information from April 1, 2012 onwards. The Discharge Abstract Database (DAD) and the National Ambulatory Care Reporting System (NACRS) database include demographic, administrative, diagnostic, procedural, and resource intensity weight (RIW) information on all patients discharged from AHS hospital and facility-based ambulatory care clinics, respectively. The diagnostic data used are International Classification of Disease—Version 10—Canadian Enhancement (ICD-10-CA) codes, and contain a most responsible diagnosis field and up to 24 secondary diagnostic codes in DAD and 9 secondary diagnostic codes in NACRS. All data were linked to the Population Registry, which contains demographic information for all Albertans with Alberta Health Care Insurance Plan (AHCIP) coverage; the AHCIP is a publicly funded, government administered insurance plan that all residents are eligible for and over 99% participate [14]. Data relating to migration in and out of the province, as well as death was used from this database to ensure completeness of data.

### Cohort selection

The population of interest were patients who received at least one dispensation for a SCIg or IVIg therapeutic product within the observation period from April 1, 2012 to March 31, 2019; see Additional File 1 for a list of the IgG therapeutic products included in this study. Index date was defined as the first date of an IgG dispensation from a blood clinic facility within the observation period. Other eligibility criteria included having continuous AHCIP coverage during the 2-year pre-index period and the years that a patient received treatment during the observation period, starting from the year within which the index date occurred through to the year a patient exited the study (i.e., did not receive an IgG dispensation within the year, left the province, or died). Clinic-administered or self-administered SCIg was identified by the presence or absence of an ambulatory care visit containing the ICD-10-CA diagnostic code Z29.1 (prophylactic immunotherapy, including the administration of immunoglobulin) within 7-days after an IgG dispensation, respectively. Patients were grouped according to treatment location (self-administered or clinic-administered) and route of IgG administration (SC or IV), as well as age (overall, adults [≥ 18-years], and children [< 18-years]); region of residence was considered (Northern and Southern Alberta).

### Characteristics

Demographic characteristics were measured on the index date and included age, sex, and residence that was categorized by urban or rural from the second digit of the postal code, as well as the northern and southern parts of the province by forward sortation area from the first three digits of postal codes [15]. The Charlson Comorbidity Index (CCI) score was determined during the 2-year pre-index period using ICD-10-CA codes of specific medical conditions weighted according to their potential for influencing mortality [16]. The specific comorbidities used within the CCI were also determined, as well as identification of subjects with a primary or secondary immunodeficiency defined as ≥ 1 hospitalization or ambulatory care visit with a corresponding diagnosis during the observation period (see Additional File 2).

### Cost determination

Costs from the perspective of the Canadian health care system were determined by measuring units of relevant health care resources utilized and determining cost per unit; costs included medical laboratory staff and nursing time, as well as ambulatory care visits. The cost of IgG, and the transportation and storage of the IgG therapeutic products prior to dispensation were assumed to be equivalent between SCIg and IVIg, and therefore not included in the analysis. Detailed in Table 1, costs were categorized into preparation and dispensation of SCIg and IVIg therapeutic products, training and support for self-administered SCIg, and ambulatory care visits for those who received clinic-administered IVIg; although clinic-administered SCIg was not a focus of this study, patients who received this type of IgG treatment were identified. Time estimates were provided by medical laboratory staff and nurses who performed the listed types of utilization, and regional variations were considered.

**Table 1:** Determination of costs for economic evaluation

Part A: health care utilization determined by time estimates and wage rates		
Type of utilization	Time estimate	Pay rate ($CDN)
*Preparation and dispensation*		
Request and receiving	0.5 min/vial	$26.86 per hour for a Medical laboratory assistant level II^a^ or $39.08 per hour for a Medical laboratory technologist level I^b^
Assigning		
Clinic-based IVIg and SCIg	2 min/vial	$39.08 per hour for a Medical laboratory technologist level I
Home-based SCIg	1 min/vial	$26.86 per hour for a Medical laboratory assistant level II^a^ or $39.08 per hour for a Medical laboratory technologist level I^b^
Quality assurance	10 min/patient	$41.61 per hour for a Medical laboratory technologist level II
Dispensation	0.5 min/vial	$26.86 per hour for a Medical laboratory assistant level II^a^ or $39.08 per hour for a Medical laboratory technologist level I^b^
Additional costs for patients in Edmonton receiving IgG at locations other than the central facility		
Shipping from central facility	0.5 min/vial	$26.86 per hour for a Medical laboratory assistant level II
Receiving at blood facility	0.5 min/vial	$39.08 per hour for a Medical laboratory technologist level I
*Training and support for self-administered SCIg (occurred the first year)*		
Training (occurred first year)		
Patients in Northern Alberta	6.75 h	$43.94 per hour for a registered nurse
Patients in Southern Alberta	9 h	$43.94 per hour for a registered nurse

Part B: health care utilization determined by number of ambulatory clinic visits and cost per visit		
Type of utilization	Number of visits	Cost per visit ($CDN)
*Ambulatory clinic visits*		
Clinic-based IVIg and SCIg	Determined from administrative data	Determined by multiplying the RIW assigned to each clinic visit for IgG administration (i.e., Z29.1) by the CIHI cost of a standard ambulatory visit in Alberta during the year within which the visit occurred

*CAD* Canadian; *CIHI* Canadian Institute for Health Information; *IVIg* Intravenous immunoglobulin G; *RIW* Resource intensity weight; *SCIg* Subcutaneous immunoglobulin G

^a^used for costing calculations among patients in the Edmonton Area

^b^used for costing calculations among patients in all other areas of the province

Total preparation and dispensation costs per patient were calculated by multiplying the total number of IVIg or SCIg vials and dispensations per patient by the cost to request and receive, assign, and dispense each vial, and perform quality assurance for each dispensation (i.e., time to perform each task multiplied by the pay rate of the medical laboratory staff member [source: AHS]) over the length of time each patient was considered to have received their respective therapy (i.e., between the first date of dispensation for a specific route/location of IgG therapy and either one month following the last clinic-administered IVIg or SCIg treatment or two months following the last SCIg dispensation for self-administered treatment among adults and one month following the last SCIg dispensation for children; based on preliminary results). Patients who initiated self-administered SCIg received two one-on-one training sessions with a registered nurse. The estimated total training time (i.e., 6.75 h for patients in Northern Alberta, and 9 h for patients in Southern Alberta) was multiplied by the hourly pay rate of a registered nurse in Alberta [17] to determine the one-time total cost of training for those who initiated self-administered SCIg during the observation period. Considering that not all the ambulatory care visits for clinic-administered IVIg and SCIg therapy were specifically coded with Z29.1 in the primary diagnostic field, the cost of clinic-administered IgG was determined by multiplying the number of ambulatory care visits for IVIg or SCIg administration with the average cost of visits that had a Z29.1 diagnostic code located in the primary field (i.e., RIW value associated with the visit multiplied by the Canadian Institute for Health Information cost of a standard ambulatory care visit [CSHS] in Alberta) [18]. RIW is a measure to estimate healthcare resource use and represents the relative value of resources that a given patient, contingent on diagnostic case-mix, would be expected to consume relative to a standard patient. The CSHS provides standardized average costs incurred through the direct care of a standard outpatient visit (e.g., nursing, diagnostic, and therapeutic costs); the cost of IgG was not included in the RIW and CSHS computation algorithm [18]. Collectively, IgG administration costs for all patients included preparation and dispensation of applicable IgG therapeutic products, training and support during the first year for those who initiated self-administered SCIg, and ambulatory care visit costs for those who received clinic-administered IVIg or SCIg therapy. Total administration costs were then divided by the number of months a patient was on a respective IgG treatment, and multiplied by twelve to calculate the per-year cost. All costs were adjusted to 2020 values using the Canadian Consumer Price Index [19].

### Incremental cost of switching to self-administered SCIg

The economic impact of switching patients in Alberta from clinic-administered IVIg to self-administered SCIg therapy was estimated based on cost estimates and use case assumption using the health care system perspective. Based on clinical expert opinion and survey findings from Reid et al. [7] who found that 78% of adult and pediatric patients in Ontario who received clinic-administered IVIg were eligible and willing to switch to self-administered SCIg [7], 2 scenario analyses were developed; a conservative scenario assumed that 50% of all patients who currently receive clinic-administered IVIg in Alberta would switch to self-administered SCIg, and an optimistic scenario assumed that 80% would switch to self-administered SCIg.

Regional variation in the use of self-administered SCIg therapy may exist in the province, as such the economic impact of patients who received clinic-administered IVIg and those who received self-administered SCIg therapy by region (Northern and Southern Alberta) was determined.

### Statistical analysis

Patient characteristics were summarized using means (± standard deviation [SD]), medians (interquartile ranges), counts, and percentages, as appropriate. Univariate generalized linear model regression with gamma distribution and log link was used to compare cost differences between the different groups (i.e., self-administered SCIg, clinic-administered IVIg, and clinic-administered SCIg). All analyses were performed using Stata version 14 (Stata Corporation, College Station, Texas) and R version 3.5.2 (R Foundation for Statistical Computing, Vienna); 2-sided p-values < 0.05 were considered statistically significant.

## Results

### Cohort

**Fig. 1:**
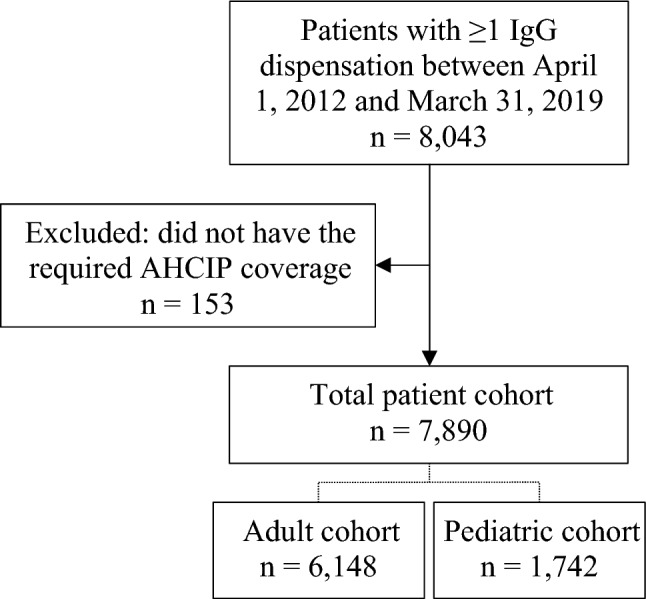
Cohort selection flowchart. Abbreviations: *AHCIP* Alberta Health Care Insurance Plan; *IgG* Immunoglobulin G

8,043 patients received at least one dispensation for an IgG therapeutic product during the observation period (Fig. 1), and after excluding 153 patients who did not have the required AHCIP coverage, the final cohort consisted of 7,890 patients, of which 78% were adults (n = 6,148) and 22% were children (n = 1,742).

### Characteristics

Baseline characteristics are shown in Table 2. Mean age was 45 (± 26) years; the mean age of the adult cohort was 56 (± 17) years and the pediatric cohort was 6 (± 5) years. Females comprised just over half of the adult cohort (52.7%) and just under half of pediatric cohort (46.6%). The vast majority of patients lived in urban areas (84.9% of all patients). While residency was equally divided between Northern (50.2%) and Southern (49.8%) Alberta among all patients, the majority of the adult cohort resided in northern Alberta (53.6%) and the majority of the pediatric cohort resided in southern Alberta (61.8%). The CCI within the adult cohort was 1.8 (± 2.3), and the most common (> 10%) CCI-specific comorbidities were cancer (18.6%), diabetes (18.5%), chronic pulmonary disease (18.0%), and rheumatoid disease (10.5%). Within the pediatric cohort, the CCI was 0.7 (± 1.7). Those with primary or secondary immunodeficiencies comprised 27.5% of the adult cohort and 10.3% of the pediatric cohort. On the index date, 95.7% of patients received clinic-administered IVIg and 4.3% received SCIg therapy, of which the vast majority received self-administered SCIg.

**Table 2:** Baseline characteristics of the patient cohort

	Total n = 7,890	Adults n = 6,148	Pediatric n = 1,742
*Demographic characteristics*			
Age, years			
Mean (SD)	45 (26)	56 (17)	6 (5)
Median (IQR)	51 (24,66)	58 (43,69)	4 (1,9)
Sex, n (%)			
Females, n (%)	4,053 (51.4%)	3,242 (52.7%)	811 (46.6%)
Residence, n (%)			
Urban	6,697 (84.9%)	5,187 (84.4%)	1,510 (86.7%)
Northern Alberta	3,960 (50.2%)	3,295 (53.6%)	665 (38.2%)
Southern Alberta	3,930 (49.8%)	2,853 (46.4%)	1,077 (61.8%)
*Clinical characteristics*			
Charlson Comorbidity Index			
Score, mean (SD)	1.6 (2.2)	1.8 (2.3)	0.7 (1.7)
Comorbidities within the index, n (%)			
Cancer	1,307 (16.6%)	1,145 (18.6%)	162 (9.3%)
Chronic pulmonary disease	1,208 (15.3%)	1,106 (18.0%)	102 (5.9%)
Diabetes	1,169 (14.8%)	1,140 (18.5%)	29 (1.7%)
Rheumatoid disease	694 (8.8%)	647 (10.5%)	47 (2.7%)
Renal disease	606 (7.7%)	557 (9.1%)	49 (2.8%)
Heart failure	514 (6.5%)	480 (7.8%)	34 (2.0%)
Liver disease	392 (5.0%)	335 (5.5%)	57 (3.2%)
Cerebrovascular disease	378 (4.8%)	335 (5.5%)	43 (2.5%)
Myocardial infarction	252 (3.2%)	> 243 (> 4.0%)	< 10
Metastatic cancer	252 (3.2%)	200 (3.3%)	52 (3.0%)
Peripheral vascular disease	210 (2.7%)	184 (3.0%)	26 (1.5%)
Hemiplegia or paraplegia	168 (2.1%)	110 (1.8%)	58 (3.3%)
Peptic ulcer	148 (1.9%)	> 139 (> 2.3%)	< 10
Dementia	76 (1.0%)	76 (1.2%)	N/A
Primary or secondary immunodeficiency, n (%)	1,872 (23.7%)	1,692 (27.5%)	180 (10.3%)
*IgG administration on the index date, n (%)*			
IVIg	7,547 (95.7%)	5,819 (94.7%)	1,728 (99.2%)
SCIg	343 (4.3%)	329 (5.3%)	14 (0.8%)
Self-administered	> 334 (> 4.2%)	> 320 (> 5.2%)	> 10 (> 0.6%)
Clinic-administered	< 10	< 10	< 10

*IQR* interquartile range; *IVIg* intravenous immunoglobulin G; *SCIg* subcutaneous immunoglobulin G; *SD* standard deviation

### Total patient population

During the observation period, 684 patients received self-administered SCIg (Northern Alberta: 502 patients; Southern Alberta: 182 patients), 7,584 received clinic-administered IVIg (Northern Alberta: 3,681 patients; Southern Alberta: 3,903 patients), and 56 received clinic-administered SCIg (Northern Alberta: 39 patients; Southern Alberta: 17 patients). Table 3 details costs overall, as well as during the first and subsequent years(s) of self-administered SCIg and clinic-administered IVIg treatment among all patients, and the adult and pediatric cohorts. See Additional File 3 for comparisons with clinic-administered SCIg.

**Table 3:** Self-administered SCIg and clinic-administered IVIg health care resource use and costs presented overall, and during the first and subsequent years(s) of treatment among the total patient population, and the adult and pediatric cohorts

	Total patient population			Adult cohort			Pediatric cohort		
	Self-administered SCIg	Clinic-administered IVIg	Increment: SCIg minus IVIg	Self-administered SCIg	Clinic-administered IVIg	Increment: SCIg minus IVIg	Self- administered SCIg	Clinic-administered IVIg	Increment: SCIg minus IVIg
Cost ($CDN) per patient-year, mean (95% CI); n or p-value									
*Overall*									
Total	$817 (723,912); n = 684	$6,204 (6100,6308); n = 7,584	-$5,386 (-5039,-5734); p < 0.001	$782 (696,868); n = 638	$6,714 (6586,6841); n = 5,852	-$5,931 (-5543,-6319); p < 0.001	$1,305 (549,2060); n = 46	$4,482 (4369,4595); n = 1,732	-$3,177 (-2473,-3882); p < 0.001
Preparation and dispensation	$237 (221,254)	$246 (242,250)		$235 (219,252)	$272 (267,277)		$261 (160,363)	$159 (154,163)	
Training (SCIg) or visits (IVIg)	$323 (320,326)	$5,958 (5857,6058)		$320 (317,323)	$6,441 (6318,6564)		$363 (349,377)	$4,323 (4214,4432)	
*First-year*									
Total	$573 (556,590); n = 684	$6,319 (6213,6424); n = 7,584	-$5,746 (-5639,-5852); p < 0.001	$569 (553,585); n = 638	$6,853 (6723,6982); n = 5,852	-$6,284 (-6153,-6414); p < 0.001	$628 (523,732); n = 46	$4,514 (4401,4628); n = 1,732	-$3,887 (-3734,-4039); p < 0.001
Preparation and dispensation	$250 (234,266)	$250 (246,254)		$249 (233,265)	$277 (272,282)		$264 (163,366)	$159 (154,163)	
Training (SCIg) or visits (IVIg)	$323 (320,326)	$6,068 (5967,6170)		$320 (317,323)	$6,575 (6451,6700)		$363 (349,377)	$4,355 (4246,4465)	
*Subsequent year(s)*									
Total	$250 (185,314); n = 406	$4,045 (3589,4501); n = 1,988	-$3,796 (-3335,-4256); p < 0.001	$254 (186,322); n = 381	$4,267 (3733,4801); n = 1,692	-$4,013 (-3475,-4552) p < 0.001	$186 (138,233); n n = 25	$2,776 (2589,2963); n = 296	-$2,591 (-2386, -2783); p < 0.001
Preparation and dispensation	$250 (185,314)	$160 (142,177)		$254 (186,322)	$170 (150,191)		$186 (138,233)	$101 (94,107)	
Training (SCIg) or visits (IVIg)	N/A	$3,885 (3,447,4,324)		N/A	$4,097 (3583,4610)		N/A	$2,675 (2495,2856)	

Univariate generalized linear model regression with gamma distribution and log link was used to compare cost differences between self-administered SCIg and clinic-administered IVIg; two-sided p-values < 0.05 were considered statistically significant

*IVIg* intravenous immunoglobulin G; *SCIg* subcutaneous immunoglobulin G

The overall mean per patient-year cost of self-administered SCIg was $817 (95% confidence interval [CI] $723, $912), which included preparation and dispensation costs while on treatment (mean 2.4 years [95% CI 2.2, 2.5]) and the initial training costs incurred in the first year; clinic-administered IVIg was $6,204 (95% CI $6,100, $6,308), which included preparation and dispensation costs while on treatment (mean 1.1 years [95% CI 1.1, 1.2]), and ambulatory care clinic visit costs over the entire treatment period. The overall mean incremental cost of self-administered SCIg was $5,386 (95% CI $5,039, $5,734; p < 0.001) less than clinic-administered IVIg per patient-year. During the first year, the mean incremental cost of self-administered SCIg ($573; 95% CI $556, $590) was $5,746 (95% CI $5,639, $5,852; p < 0.001) less than clinic-administered IVIg ($6,319; 95% CI $6,213, $6424), and during subsequent year(s), self-administered SCIg ($250; 95% CI $185, $314) was $3,796 (95% CI: $3,335, $4256; p < 0.001) less than clinic-administered IVIg ($4,045; 95% CI $3,589, $4,501) per patient-year. Additional File 4 details the overall mean per-patient year cost of self-administered SCIg and clinic-administered IVIg within Northern and Southern Alberta.

### Adult cohort

During the time that adult patients received IgG treatment (self-administered SCIg: 2.4 years, 95% CI 2.3, 2.6; clinic-administered IVIg: 1.2 years, 95% CI 1.2, 1.3), self-administered SCIg treatment cost an average of $782 (95% CI $696, $868) per patient-year, and clinic-administered IVIg treatment was $6,714 (95% CI $6,586, $6,841). Self-administered SCIg was $5,931 (95%CI $5543, $6319; p < 0.001) less than clinic-administered IVIg per patient-year on average. During the first year, self-administered SCIg ($569; 95% CI $553, $585) was $6,284 (95% CI $6153, $6414; p < 0.001) less than clinic-administered IVIg ($6,853; 95% CI $6723, $6982) per patient-year. During the subsequent year(s), self-administered SCIg ($254; 95% CI $186, $322) was $4,013 (95% CI: $3,475, $4,552; p < 0.001) less than clinic-administered IVIg ($4,267; 95% CI $3733, $4801) per patient-year.

### Pediatric cohort

During the time that pediatric patients received IgG treatment (self-administered SCIg: 1.7 years, 95% CI 1.2, 2.1; clinic-administered IVIg: 0.7 years, 95% CI 0.7, 0.8), the overall mean per patient-year cost of self-administered SCIg was $1,305 (95% CI $549, $2,060) and clinic-administered IVIg was $4,482 (95% CI $4,369, $4,595). Self-administered SCIg was $3,177 (95% CI $2,473, $3,882; p < 0.001) less than clinic-administered IVIg per patient-year. During the first year of therapy, self-administered SCIg ($628; 95% CI $523, $732) was $3,887 (95% CI $3,734, $4,039; p < 0.001) less than clinic-administered IVIg ($4,514; 95% CI $4,401, $4,628) per patient-year. During the subsequent year(s), self-administered SCIg ($186; 95% CI $138, $233) was $2,591 (95% CI: $2,386, $2,783; p < 0.001) less than clinic-administered IVIg ($2,776; 95% CI $2,589, $2,963) per patient-year.

### Estimated incremental cost of switching to self-administered SCIg

During the observation period, 91% of the total patient population only received clinic-administered IVIg. Therefore, a total of 7,199 patients were considered in the analytic scenarios. Considering that self-administered SCIg was $5,386 (95% CI $5,039, $5,734) less costly overall compared with clinic-administered IVIg per patient-year among the total patient population, in a scenario of 50% (n = 3,600) of patients switching from clinic-administered IVIg to self-administered SCIg, a $19.4 million (95% CI $18.1, $20.6) reduction in cost was estimated over the observation period.^1^ If the proportion of patients who switched to self-administered SCIg was increased to 80% (n = 5,759), a $31.0 million (95% CI $29.0, $33.0) cost reduction was estimated.^2^ The overall cost difference estimates accounted for the training involved in initiating self-administered SCIg treatment, which was found to be an average of $323 (95% CI $320, $326) in nursing time per patient. Therefore, the one-time cost of training to switch 3,600 patients from clinic-administered IVIg to self-administered SCIg was estimated to be $1.2 million (95% CI $1.2, $1.2) in nursing time, and $1.9 million (95% CI $1.8, $1.9) if 5,759 patients were switched. Accounting for regional differences in patients who only received clinic-administered IVIg during the observation period and the per patient-year incremental cost difference between self-administered SCIg and clinic-administered IVIg, the cost savings if self-administered SCIg use was increased to 50% was estimated to be $8.8 (95% CI $8.1, $9.5) and $10.0 (95% CI $8.7, $11.2) million in Northern and Southern Alberta, respectively (see Additional File 5 for detailed results).

## Discussion

In this retrospective, administrative health data population-based cohort study of 6148 adults and 1742 children who received IgG therapy between April 1, 2012 and March 31, 2019 in Alberta, Canada, a comparative economic evaluation of SCIg and IVIg within home and clinic settings was performed, and estimates of the potential health care cost difference if various proportions of patients transitioned from clinic-administered IVIg to self-administered SCIg therapy was conducted. A cost analysis determined that overall, the average incremental cost of self-administered SCIg was $5,386 lower compared with clinic-administered IVIg per patient-year ($817 versus $6,204) among all patients; self-administered SCIg was $5,931 lower compared with clinic-administered IVIg ($782 versus $6,714) among adults and $3,177 lower ($1,305 versus $4,482) among children, per patient-year. It was estimated that an average of $19.4 million in health care system cost savings, comprised of medical laboratory staff and nursing time, and ambulatory care clinic visits, could have been realised if 50% of patients who only received clinic-administered IVIg treatment in this study switched to self-administered SCIg, and $31.0 million saved if 80% switched. Regional differences in current IgG treatment practice were observed.

Economic evaluations conducted in Canada have included various types of direct costs to the health care system such as nursing and technologist time, outpatient charges (hospital or clinic), physician visits, overhead, IgG products, and/or infusion supplies when comparing self-administered SCIg with clinic-administered IVIg [5, 8–13]. Regardless of the included costs, these studies have shown that self-administered SCIg was less costly compared with clinic-administered IVIg, primarily because of less nursing and outpatient costs [5, 8]. In the current study, the costs of medical laboratory staff and nursing time, as well as ambulatory care visits were included, which reflects current IgG administration practices within the Alberta health care system; the cost of self-administered SCIg infusion supplies were not included as these are provided to the patient by the manufacturer. Similar to other reports [8, 9, 13], the cost of IgG products was not included in our analysis, although some previous economic evaluations have incorporated this cost [5, 10–12]. While IgG product costs have been shown to account for the largest proportion of the overall direct cost of treatment (> 85% in some cases) [5, 10–12], the incremental cost difference between clinic-administered IVIg and self-administered SCIg treatment is not appreciably affected by the cost of the IgG products themselves in Canada; there is parity in the price per gram of SCIg and IVIg [11], economic modeling studies have considered the per patient cost of SCIg and IVIg products to be equivalent [5, 10, 11], and the directly measured dose (and resultant cost) of SCIg and IVIg products has been shown to be not significantly different among treated patients with primary immunodeficiency [12]. It should be noted that this is not necessarily the case in all health care systems. For example, in the United States the price per gram of SCIg products are generally priced higher than IVIg, and a higher dose of SCIg is recommended [20, 21].

Considering that initiation of self-administered SCIg requires training, some modeling studies have incorporated this cost into the first year of self-administered SCIg treatment while assuming consistent costs for clinic-administered IVIg over time [5, 8, 9]. Using the average annual wage and benefits of registered nurses in British Columbia in 2011, [9]) applied this convention to estimate the potential difference in nursing costs if patients with primary and secondary immunodeficiencies were switched from clinic-administered IVIg to self-administered SCIg [9]. The authors estimated that the expenditure required for nursing time in the first year would be $691 (12 h of training and monitoring) and $345 (6 h of monitoring) annually thereafter for self-administered SCIg to offset the $3,294 of clinic nursing time annually for IVIg, resulting in an estimated economic gain of $2,603 in the first year, and $2,948 in each subsequent year of treatment from switching one patient to self-administered SCIg [9]. Although we found that the average cost of initiating self-administered SCIg was greater in the first year ($573 per patient-year) than subsequent year(s) ($250 per patient-year), primarily due to the nursing time required for training in the first year, we also found that the average cost of clinic-administered IVIg was not consistent over time. Among patients who initiated clinic-administered IVIg, the mean cost in the first year was $6,319 ($250 for preparation and dispensation, and $6,068 for ambulatory care clinic visits) per patient, and $4,045 ($160 for preparation and dispensation, and $3,885 for ambulatory care clinic visits) per patient-year in subsequent year(s); this decrease in cost appears to be driven by a decrease in the number of clinic visits for IVIg over time. In Alberta, guidelines for the clinical use of IgG recommends that for most chronic conditions, a reduction in IgG dose and/or treatment interval to the lowest clinically effective dose be undertaken for maintenance therapy in stable patients [22].

The listed indications for IgG continue to increase, including off-label use. Canadian Blood Services reports that IVIg and SCIg are often used off-label and account for a significant proportion of IgG use in most Canadian provinces. Constantine *et al.* [2] found that 55% of patients received IVIg for an off-label use in the Atlantic provinces between 2003 and 2005 [2]. Therefore, in the current study, all 7199 patients who only received clinic-administered IVIg during the observation period, regardless of indication, were evaluated in the scenario analysis that was based on real world patient users of IgG therapy. The impact of switching 50% of these patients from clinic-administered IVIg to self-administered SCIg was found to be an estimated reduction of $19.4 million over the 7-year observation period, and an estimated $31.0 million may have been realised if 80% switched to self-administered SCIg.

This study has several important strengths, including the large size and population-based design that allowed for the identification of 99% of incoming and outgoing IgG products to blood clinic facilities in Alberta, and linking to provincial administrative data, including health care costs for ambulatory care clinic visits. However, results should be considered within the context of study assumptions and limitations. An overarching assumption within this economic evaluation is that SCIg and IVIg have comparable effectiveness, which has been demonstrated by others [4]. Also, the cost of IgG products, and the transportation and storage of the products prior to dispensation were assumed to be equivalent between SCIg and IVIg. The preparation and dispensation of IgG products, and training for self-administered SCIg therapy was not directly measured, and therefore time estimates were used that were provided by medical laboratory staff and nurses who performed these tasks. Although this study was conducted from the perspective of the Canadian health care system and did not include costs borne by patients or indirect costs such as productivity loss, previous studies have shown that when the societal perspective is considered, the cost savings of self-administered SCIg is even greater compared with clinic-administered IVIg [5, 10].

## Conclusions

In summary, this study found that self-administered SCIg treatment was $5,386 less costly per patient-year compared with clinic-administered IVIg among all patients ($5,931 less costly among adults, and $3,177 less costly among children per patient-year), representing a $19.4 million cost savings if 50% of patients who only received clinic-administered IVIg switched to self-administered SCIg during the 7-year observation period. This study adds to previous published evidence that self-administered SCIg is associated with better health related quality of life and higher treatment satisfaction [4, 5] by quantifying health care system savings with SCIg compared with IVIg. Therefore, switching to self-administered SCIg has potential system and patient benefits such as reducing overall health care costs in Canada, lessening nursing burden, increasing clinic capacity for others, and improving the quality of life for patients.

## Supplementary Information

**Additional file 1: **IgG therapeutic products that were included in the.

**Additional file 2: **Description of the ICD-10-CA codes used in the identification of primary and secondary immunodeficiencies.

**Additional file 3: **Clinic-administered SCIg was compared with self-administered SCIg and clinic-administered IVIg overall, and during the first and subsequent year(s) of treatment among the total patient population, and the adult and pediatric cohorts.

**Additional file 4: **Self-administered SCIg and clinic-administered IVIg health care resource use and costs presented overall among the total patient population in Northern and Southern Alberta.

**Additional file 5: **Estimated incremental cost scenarios in Northern and Southern Alberta.

## Data Availability

The data that support the findings of this study are not available. The data custodians, Alberta Health Services and Alberta Health do not allow users of the data to publish the data.

## Abbreviations

AHCIP: Alberta Health Care Insurance Plan
AHS: Alberta Health Services
CCI: Charlson Comorbidity Index
CSHS: Cost of a standard hospital stay
DAD: Discharge Abstract Database
ICD-1-CA: International Classification of Disease—Version 10—Canadian Enhancement
IgG: Immunoglobulin G
IVIg: Intravenous immunoglobulin G
NACRS: National Ambulatory Care Reporting System
RIW: Resource intensity weight
SCIg: Subcutaneous immunoglobulin G

## Footnotes

1 Calculation: 3,600 patients * $5,386 cost savings per patient = $19.4 million.

2 Calculation: 5,759 patients * $5,386 cost savings per patient = $31.0 million.
