# Supplementary material for: Economic impact of self-administered subcutaneous versus clinic-administered intravenous immunoglobulin G therapy in Alberta, Canada: a population-based cohort study
Source: Allergy Asthma Clin Immunol. 2022 Nov 24;18:99. doi: 10.1186/s13223-022-00735-6 (PMC9700869; doi:10.1186/s13223-022-00735-6)
Supplement: Supplementary file 1 — Additional file 1: IgG therapeutic products that were included in the. [file 13223_2022_735_MOESM1_ESM.docx]

Additional File 1. IgG therapeutic products that were included in the.

| Intravenous route of administration | Subcutaneous route of administration |
| --- | --- |
| Gammagard Liquid | Cuvitru |
| Gammagard S/D | Gamunex |
| Gamunex | Hizentra |
| Igivnex | Igivnex |
| IV Immune Globulin | SC Immune Globulin |
| Iveegam Immuno 5000mg (IV) |  |
| Ivigex |  |
| Octagam |  |
| Panzyga |  |
| Privigen |  |
