# Supplementary material for: Economic impact of self-administered subcutaneous versus clinic-administered intravenous immunoglobulin G therapy in Alberta, Canada: a population-based cohort study
Source: Allergy Asthma Clin Immunol. 2022 Nov 24;18:99. doi: 10.1186/s13223-022-00735-6 (PMC9700869; doi:10.1186/s13223-022-00735-6)
Supplement: Supplementary file 2 — Additional file 2: Description of the ICD-10-CA codes used in the identification of primary and secondary immunodeficiencies. [file 13223_2022_735_MOESM2_ESM.docx]

Additional File 2. Description of the ICD-10-CA codes used in the identification of primary and secondary immunodeficiencies.

| Description | ICD-10-CA code |
| --- | --- |
| Immunodeficiency with predominantly antibody defects | D80 |
| Combined immunodeficiencies | D81 |
| Immunodeficiency associated with other major defects | D82 |
| Common variable immunodeficiency | D83 |
| Other immunodeficiencies | D84 |

ICD-10-CA = International Classification of Disease - Version 10 - Canadian Enhancement. The code may occur in any diagnostic field of an ambulatory or hospitalization encounter.
