# Supplementary material for: Economic impact of self-administered subcutaneous versus clinic-administered intravenous immunoglobulin G therapy in Alberta, Canada: a population-based cohort study
Source: Allergy Asthma Clin Immunol. 2022 Nov 24;18:99. doi: 10.1186/s13223-022-00735-6 (PMC9700869; doi:10.1186/s13223-022-00735-6)
Supplement: Supplementary file 3 — Additional file 3: Clinic-administered SCIg was compared with self-administered SCIg and clinic-administered IVIg overall, and during the first and subsequent year(s) of treatment among the total patient population, and the adult and pediatric cohorts. [file 13223_2022_735_MOESM3_ESM.docx]

Additional File 3. Clinic-administered SCIg was compared with self-administered SCIg and clinic-administered IVIg overall, and during the first and subsequent year(s) of treatment among the total patient population, and the adult and pediatric cohorts.

|  | Total patient population | | |  | Adult Cohort | | |  | Pediatric Cohort | | | |
| --- | --- | --- | --- | --- | --- | --- | --- | --- | --- | --- | --- | --- |
|  | Clinic-administered  SCIg | Self-administered  SCIg | Clinic-administered IVIg |  | Clinic-administered  SCIg | Self-administered  SCIg | Clinic-administered IVIg |  | Clinic-administered  SCIg | Self-administered  SCIg | | Clinic-administered IVIg |
| Average cost ($CDN) per patient-year, mean (95% CI); n | | | | | | | | | | | | |
| **Overall** |  |  |  |  |  |  |  |  |  |  |  |  |
| Total | $5,089^a^  (3761,6417);  n=56 | $817  (723,912);  n=684 | $6,204  (6100,6308);  n=7,584 |  | $4,617^a,b^  (4027,5208);  n=>46 | $782  (696,868);  n=638 | $6,714  (6586,6841);  n=5,852 |  | N/A*  (N/A);  n<10 | $1,305  (549,2060);  n=46 | | $4,482  (4369,4595);  n=1,732 |
|  |  |  |  |  |  |  |  |  |  |  |  |  |
| Preparation and dispensation | $540  (400,680) | $237  (221,254) | $246  (242,250) |  | $562  (406,718) | $235  (219,252) | $272  (267,277) |  | N/A | $261  (160,363) | | $159  (154,163) |
|  |  |  |  |  |  |  |  |  |  |  |  |  |
| Training or  clinic visits | $4,549  (3248,5850) | $323  (320,326) | $5,958  (5857,6058) |  | $4,055  (3482,4629) | $320  (317,323) | $6,441  (6318,6564) |  | N/A | $363  (349,377) | | $4,323  (4214,4432) |
|  |  |  |  |  |  |  |  |  |  |  |  |  |
| **First-year** |  |  |  |  |  |  |  |  |  |  |  |  |
| Total | $4,896^a,b^  (3563,6229);  n=56 | $573  (556,590);  n=684 | $6,319  (6213,6424);  n=7,584 |  | $4,396^a,b^  (3806,4987);  n=>46 | $569  (553,585);  n=638 | $6,853  (6723,6982);  n=5,852 |  | N/A*  (N/A);  n<10 | $628  (523,732);  n=46 | | $4,514  (4401,4628);  n=1,732 |
|  |  |  |  |  |  |  |  |  |  |  |  |  |
| Preparation and dispensation | $531 (389,672) | $250  (234,266) | $250  (246,254) |  | $551 (393,709) | $249 (233,265) | $277 (272,282) |  | N/A | $264 (163,366) | | $159  (154,163) |
|  |  |  |  |  |  |  |  |  |  |  |  |  |
| Training or  clinic visit | $4,365 (3063-5668) | $323  (320,326) | $6,068  (5967,6170) |  | $3,845 (3282-4409) | $320  (317,323) | $6,575 (6451,6700) |  | N/A | $363 (349,377) | | $4,355 (4246-4465) |
|  |  |  |  |  |  |  |  |  |  |  |  |  |
|  |  |  |  |  |  |  |  |  |  |  | |  |
| **Subsequent year(s)** |  |  |  |  |  |  |  |  |  |  | |  |
| Total | N/A*  (N/A);  n<10 | $250  (185,314);  n=406 | $4,045  (3589,4501);  n=1,988 |  | N/A*  (N/A);  n<10 | $254  (186,322);  n=381 | $4,267  (3733,4801);  n=1,692 |  | N/A*  (N/A);  n=0 | $186  (138,233);  n=25 | | $2,776  (2589-2963);  n=296 |
|  |  |  |  |  |  |  |  |  |  |  |  |  |
| Preparation and dispensation | N/A | $250  (185,314) | $160  (142,177) |  | N/A | $254  (186,322) | $170 (150,191) |  | N/A | $186  (138,233) | | $101  (94,107) |
|  |  |  |  |  |  |  |  |  |  |  |  |  |
| Training or  clinic visit | N/A | N/A | $3,885  (3,447,4,324) |  | N/A | N/A | $4,097 (3583-4610) |  | N/A | N/A | | $2,675 (2495-2856) |

Univariate generalized linear model regression with gamma distribution and log link was used to compare cost differences; two-sided p-values <0.05 were considered statistically significant. Statistically significantly different compared with ^a^self-administered SCIg, ^b^clinic-administered IVIg. *Statistical comparisons with clinic-administered SCIg were not performed due to the small group size. CI = confidence interval; IVIg = intravenous immunoglobulin G; SCIg = subcutaneous immunoglobulin; N/A = not applicable.
