# Supplementary material for: Economic impact of self-administered subcutaneous versus clinic-administered intravenous immunoglobulin G therapy in Alberta, Canada: a population-based cohort study
Source: Allergy Asthma Clin Immunol. 2022 Nov 24;18:99. doi: 10.1186/s13223-022-00735-6 (PMC9700869; doi:10.1186/s13223-022-00735-6)
Supplement: Supplementary file 4 — Additional file 4: Self-administered SCIg and clinic-administered IVIg health care resource use and costs presented overall among the total patient population in Northern and Southern Alberta. [file 13223_2022_735_MOESM4_ESM.docx]

Additional File 4. Self-administered SCIg and clinic-administered IVIg health care resource use and costs presented overall among the total patient population in Northern and Southern Alberta.

|  | Northern Alberta | | |  | Southern Alberta | | |
| --- | --- | --- | --- | --- | --- | --- | --- |
|  | Self-administered  SCIg | Clinic-administered  IVIg | Increment:  SCIg minus IVIg |  | Self-administered  SCIg | Clinic-administered  IVIg | Increment:  SCIg minus IVIg |
| Time on treatment, mean years (95% CI) | 2.7 (2.5,2.9) | 1.1 (1.0,1.2) |  |  | 1.6 (1.4,1.8) | 1.1 (1.1,1.2) |  |
| Cost ($CDN) per patient-year, mean (95% CI); n or p-value | | | | | | |  |
| *Overall* | | | | | | |  |
| Total | $635  (577,694);  n=502 | $5,744  (5595,5893);  n=3,681 | -$5,109  (-5,512,-4,705);  P<0.001 |  | $1,320  (1012,1627);  n=182 | $6,638  (6493,6782);  n=3,903 | -$5,318  (-5,990,-4,647);  P<0.001 |
|  |  |  |  |  |  |  |  |
| Preparation and dispensation | $224  (209,239) | $229  (223,234) |  |  | $274  (227,320) | $263  (257,268) |  |
|  |  |  |  |  |  |  |  |
| Training (SCIg) or visits (IVIg) | $297  (N/A) | $5,515  (5372,5659) |  |  | $395  (N/A) | $6,375  (6236,6514) |  |
|  |  |  |  |  |  |  |  |

Univariate generalized linear model regression with gamma distribution and log link was used to compare cost differences; two-sided p-values <0.05 were considered statistically significant. Abbreviations: IVIg – intravenous immunoglobulin G; N/A – not applicable; SCIg – subcutaneous immunoglobulin G.
