# Supplementary material for: Economic impact of self-administered subcutaneous versus clinic-administered intravenous immunoglobulin G therapy in Alberta, Canada: a population-based cohort study
Source: Allergy Asthma Clin Immunol. 2022 Nov 24;18:99. doi: 10.1186/s13223-022-00735-6 (PMC9700869; doi:10.1186/s13223-022-00735-6)
Supplement: Supplementary file 5 — Additional file 5: Estimated incremental cost scenarios in Northern and Southern Alberta. [file 13223_2022_735_MOESM5_ESM.docx]

Additional File 5. Estimated incremental cost scenarios in Northern and Southern Alberta.

A) Scenario: incremental cost savings of patients who received SCIg during the observation period.

Northern Alberta

Calculation: 502 patients * $5,109 cost savings per patient = $2.6 million (95% CI 2.4,2.8)

Southern Alberta

Calculation: 182 patients * $5,318 cost savings per patient = $1.0 million (95% CI 0.8,1.1)

B) Scenario: estimated incremental cost of switching 50% of patients who received IVIg only during the observation period to SCIg.

Northern Alberta: 3,452 patients with IVIg only

Calculation: 1,726 * $5,109 cost savings per patient = $8.8 million (95% CI 8.1,9.5)

Southern Alberta: 3,747 patients with IVIg only

Calculation: 1,874 * $5,318 cost savings per patient = $10 million (95% CI 8.7,11.2)
